# The Effectiveness of Self-Esteem-Related Interventions in Reducing Suicidal Behaviors: A Systematic Review and Meta-Analysis

**DOI:** 10.3389/fpsyt.2022.925423

**Published:** 2022-06-15

**Authors:** Nguyen Tan Dat, Nobuyuki Mitsui, Satoshi Asakura, Keisuke Takanobu, Yutaka Fujii, Kuniyoshi Toyoshima, Yuki Kako, Ichiro Kusumi

**Affiliations:** ^1^Department of Psychiatry, Hokkaido University Graduate School of Medicine, Sapporo, Japan; ^2^The Department of Psychiatry, Health Care Center of Hokkaido University, Sapporo, Japan

**Keywords:** self-esteem, psychological interventions, suicide, suicidal ideation, suicide prevention

## Abstract

Suicide is a serious social issue and is often treated using psychological interventions. The current systematic review and meta-analysis aimed to investigate the effectiveness of self-esteem-related interventions on suicidal behaviors. A systematic literature search for randomized controlled trials (RCTs) including a self-esteem component was conducted on 29 May 2021 and updated on 4 April 2022. In total, 12 studies were included in the systematic review and five studies were included in the meta-analysis. Small effect sizes were found for suicidal ideation at post intervention [*g* = −0.24, 95% CI (−0.48, 0.00)] and a 3-month follow-up [*g* = −0.36, 95% CI (−0.62, −0.11)]. However, these results should be interpreted cautiously due to the limited number of included studies and varied sample population. In conclusion, the current review suggests that future intervention studies should incorporate self-esteem enhancement in the treatment of suicidal behaviors, especially for suicidal ideation.

**Systematic Review Registration:**

https://www.crd.york.ac.uk/prospero/display_record.php?RecordID=250882

## Introduction

Suicide is seen as a global phenomenon. The World Health Organization reported that approximately 800,000 suicide deaths occur per year, which means that one person dies by committing suicide every 40 s (1). Suicide is considered a serious social issue because it not only affects the deceased person but also leaves a tremendous impact on suicide survivors and the economy. In terms of its social effects, a recent study in United States demonstrated that a single suicide death can affect up to 135 people (2). Additionally, family members and close acquaintances left behind by the suicide victim usually experience social withdrawal, guilt, self-blame, mental disorders and are even at risk of committing suicide (2, 3). In terms of the economic consequences, suicide death is suggested to lead to a loss of productivity from those engaging in suicidal behaviors and the family members left behind (4). Thus, more research into intervention programs to prevent future suicide is imperative.

A meta-analysis of RCTs from the last five decades revealed that while interventions were effective in reducing suicidal thoughts and behaviors, the effect size was small across all studies (5). This result was similarly replicated in another review using the adolescent population over 26 years (6). These studies suggested that future suicide intervention research should use different approach and focus on targeting the underlying mechanism of suicide (5, 6).

One possible mechanism for suicide is self-esteem. Low self-esteem is associated with negative mental health consequences and has been linked with 21 different disorders in the Diagnostic & Statistical Manual of Mental Disorders (DSM-5) as diagnostic criteria, associated features, risk factors or consequences (7). On the other hand, high self-esteem has been shown to predict positive mental well-being, including higher levels of happiness (8), life satisfaction (9) and self-enhancement tendencies (10). A unique characteristic of self-esteem is that it is susceptible to change, especially among children and adolescents (11, 12). Thus, due to the strong association between self-esteem and mental health outcomes, and the malleable nature of self-esteem, self-esteem-based interventions might potentially be able to improve mental health (13).

The relationship between self-esteem and suicide has been researched extensively in the past few decades. Low self-esteem has been identified as one of the most significant risk factors for suicide risk and suicidal behaviors (14–17). Furthermore, self-esteem was shown to have a unique relationship with suicidal ideation beyond what could be explained by depression and hopelessness, which are two of the most common risk factors for suicide (14). In another study, low self-esteem in childhood was suggested to be a significant risk factor for the development of suicidal ideation in young adulthood (18). In contrast, high levels of self-esteem have been indicated to mitigate suicide risk (19). Thus, increasing self-esteem has been suggested as an effective treatment against suicide-related behaviors (20–22).

So far, only one meta-analysis has explored the relationship between self-esteem and suicide (23); it reported that low self-esteem is a significant risk factor for suicide attempts in youth. Additionally, there are several reviews that attempted to examine the effectiveness of self-esteem interventions but with some limitations (7, 11, 24, 25). First, two reviews (11, 25) defined self-esteem interventions as interventions comprising an outcome measure of self-esteem. While this inclusion criterion could increase the number of included studies, this is problematic as it is likely that self-esteem enhancement happened incidentally in the included studies and not as one of the treatment targets. Furthermore, it could make it difficult to determine whether changes in self-esteem in fact lead to better treatment outcomes. Second, three reviews (11, 24, 25) focused only on the effectiveness of self-esteem interventions on the self-esteem outcome while ignoring other psychological outcomes. Since self-esteem interventions are likely to increase self-esteem, it is beneficial to also consider how the interventions improve other psychological outcomes. Kolubinski et al.'s (7) review is the only exception that did not suffer from the above-mentioned issues. They suggested that a CBT treatment based on Fennell's cognitive model of low self-esteem can significantly improve self-esteem and may also be effective against depression. Notably, to date, no systematic review or meta-analysis has investigated the effectiveness of treatment focused on self-esteem in suicide prevention.

In summary, previous literature demonstrates that self-esteem is an important factor in the development of suicidal behaviors. Despite this evidence, the effectiveness of self-esteem-based interventions in prevention of suicidal behaviors remains unclear. Thus, the present review and meta-analysis aims to investigate whether treatment incorporating self-esteem enhancement is an efficacious approach to suicide prevention. This review distinguishes itself from previous reviews in several ways. First, we include only those studies that incorporate self-esteem as a treatment target in the intervention program, regardless of whether or not self-esteem is measured as an outcome. Thus, we call these types of interventions self-esteem-related interventions instead of self-esteem interventions like previous reviews. Second, the current review includes only those studies that have a suicidal outcome, such as suicidal ideation, suicide plan, or non-suicidal self-injury (NSSI). While NSSI and other suicidal behaviors are often differentiated, previous studies and theories have also shown that these two types of behaviors can often co-exist. For example, the role of NSSI is emphasized in Joiner's theory of suicide, which states that NSSI could directly increase an individual's acquired capability for suicide by making them accustomed to fear and pain (26).

This review and meta-analysis have two aims:

(1) To investigate the types of treatment or methods used to enhance self-esteem in suicide prevention.
(2) To evaluate the effectiveness of interventions that incorporate self-esteem enhancement for reducing suicidal behaviors.

## Methods

This review and meta-analysis were conducted line with the PRISMA recommendations (27) for reporting systematic reviews and meta-analyses. The protocol for this review was registered with the PROSPERO database, registration number CRD42021250882. The research strategy was developed following the Patient, Intervention, Comparison and Outcome (PICO) guideline. In the current review, patients were those who experienced suicidal behaviors and participated in a suicide prevention program using a self-esteem-related intervention. The target intervention was any psychological intervention that incorporated a self-esteem component. Self-esteem is defined in the present study as a “positive or negative attitude toward a particular object, namely, the self” [(28) p. 30]. Following this definition, interventions that included other related self-concept (e.g.: self-worth, self-criticism or self-image) in its treatment program were also included in this review. The comparison group was not limited or specified, and any type of comparison could be included. For outcomes, selected studies must include measures of suicidal thoughts and behaviors.

### Literature Search

Search terms were developed to identify studies that assessed the effectiveness of self-esteem-related psychological interventions in reducing suicidal behaviors. The key search terms included: (self-esteem or self-concept or self-perception^*^ or self-identit^*^ or self-crit^*^ or self-attack^*^ or self-worth or self-efficacy or self-image) and (intervention^*^ or program^*^ or lesson^*^ or treatment^*^ or psychotherapy or psychoeducation) and (suicid^*^). These terms were searched in the PsycINFO, PubMed and Web of Science databases for publications from inception to 29 May 2021. In addition, the clinical trial register database (www.clinicaltrials.gov) was searched to detect additional studies that had not yet been published. Similar reviews investigating the effectiveness of self-esteem interventions were also scanned for related studies. Finally, backward and forward reference searching was conducted using the included studies. In the PsycINFO and PubMed databases, Medical Subject Headings or MeSH terms were also added (see Appendix A for the full search strategy). The search was performed by the first and second authors. A second database search was undertaken on 4 April 2022 by the first author; no new article was found.

### Selection of Studies

Studies were included if they met all the following criteria

(a) Stated that one of the main targets is to enhance self-esteem (or related self-concepts)
(b) Reported suicide-related outcomes. Suicide-related outcome here includes (1) suicidal ideation, defined as the thoughts about engaging in suicidal behaviors; (2) suicide attempt, defined as an act which leads to a non-fatal outcome but with an intention die; (3) suicide plan, defined as a serious planning about how one would kill oneself; and (4) NSSI, defined as the deliberate, self-inflicted destruction of body tissue without the intention of suicide
(c) Used a randomized controlled trial (RCT) study design
(d) Published in a peer-reviewed journal
(e) Full text is available in English

Studies were excluded if they met any of the following criteria:

(a) Were not intervention studies (e.g., case studies, qualitative studies).
(b) Did not include a self-esteem component in the intervention program
(c) Did not report the assessment of a suicide-related outcome.
(d) Did not follow an RCT study design.
(e) Not published in a peer-reviewed journal (e.g., dissertation)
(f) Full text is not available in English

### Data Extraction

The following information was extracted from the articles to a spreadsheet: author names, year of publication, intervention design, duration, sample size, setting, diagnostic criteria, mean age and gender composition, country, co-morbidity, control group, number of participants in each group, outcome measures on suicidal behaviors, and outcome assessment timepoints. Data extraction was conducted by the first author and checked by the second author. When required data were unavailable, we emailed the corresponding author of the concerned article and requested access to missing data.

### Risk of Bias

Cochrane Collaboration's tool for assessing risk of bias in randomized trials (29) was used in the current review, which assessed the following domains: (a) sequence generation; (b) allocation concealment; (c) blinding of participants, personnel, and outcome assessors for each outcome; (d) incomplete outcome data; and (e) selective outcome reporting. It has been suggested that an overall judgment about the level of bias should not be made and a specific score should not be assigned, as different forms of bias are likely to be relevant depending on the nature of the research (29). Therefore, risk of bias for all criteria was reported individually in the current review.

### Data Synthesis and Analysis

The included studies were synthesized and summarized narratively. Studies suitable for meta-analysis were included and analyzed using Review Manager (RevMan, version 5.4).

Meta-analysis was conducted to determine the effectiveness of interventions on suicidal outcomes. Standardized mean difference (SMD) was calculated due to the differences in the measurement scale. The SMD was calculated for post intervention and follow-up using Hedges' adjusted g. At post intervention, SMD was calculated for suicidal ideation. Follow-up data were sparse and varied in follow-up duration (ranging from 4 weeks to 6 months). However, four studies reported results for suicidal ideation at 3-month follow-up; thus, these data were also included in the meta-analysis. Meta-analysis was conducted using a random-effect model as opposed to a fixed-effect model because studies used a wide range of interventions and designs.

*I*^2^ was calculated to assess for heterogeneity in treatment effects. According to Cochran's guideline (29), *I*^2^ can be interpreted as non-significant heterogeneity (0−40%); moderate heterogeneity (30−60%); substantial heterogeneity (50−90%) and considerable heterogeneity (75−100%).

## Results

### Studies Included in the Review

**Figure 1:**
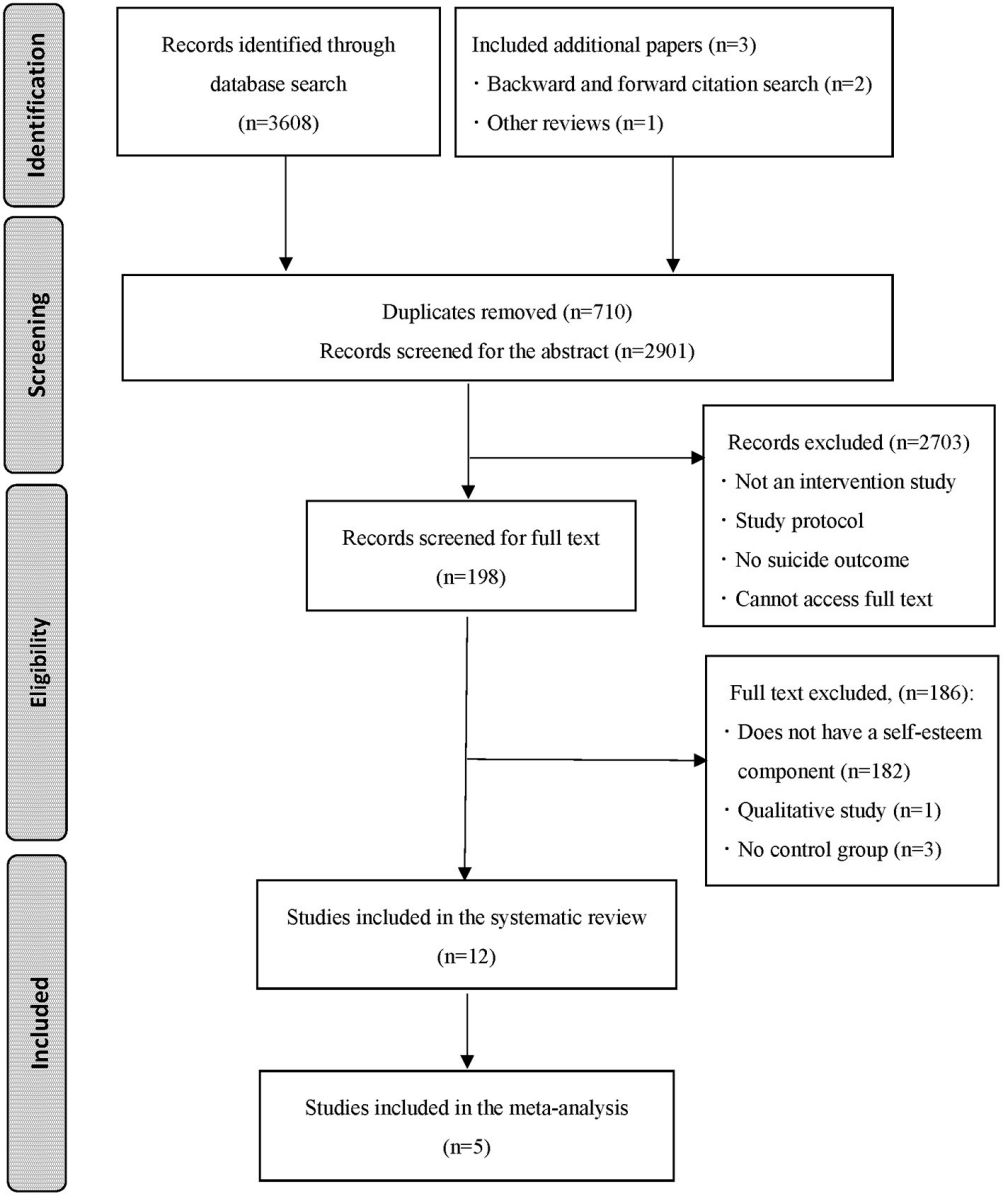
PRISMA flowchart for the process of study selection.

Figure 1 illustrates the PRISMA flowchart for the study selection process. The database search identified 3,608 articles (2,898 articles after removing duplicates). Next, titles and abstracts were screened by the first author (NTD) to determine their suitability. Through this initial check, 195 papers were selected for screening of full text by the first and second authors (NTD, MN). Disagreements between the authors were discussed until consensus was reached. A total of nine studies were found to meet the inclusion criteria after screening of the full text. Additionally, two studies (30, 31) were identified from (a) backward citation searching of an included study (32) and (b) forward citation searching of an included study (33). An additional study (34) was found through a scan of a similar systematic review (24). Thus, a total of 12 papers were included in this review.

### Study Characteristics

The total number of participants in the studies was 1,391. The sample sizes at baseline ranged from 17 to 341 (*M* = 100.35). One study (35) used an all-male sample, and two studies (33, 36) did not report the gender distribution of the sample. When studies that used an all-male sample or did not report the gender distribution of the sample were removed, the average percentage of females for all studies was 66.52%. Nine studies were conducted in the United States (30–32, 34, 36–40), one in the United Kingdom (35), one in Korea (41) and one in Australia (33). The mean age of the samples ranged from 15.42 to 51.86 years, with four studies (36–38, 40) using child or adolescent samples (aged under 18 years), four (31, 32, 34, 39) using young adult samples (aged 18–25) and four (30, 33, 35, 41) using adult samples (aged over 25 years). One study (36) did not report the mean age of the sample but stated that it comprised student in Grade 9–12. Detailed information on sample demographics and study population is given in Table 1.

**Table 1:** Sample characteristics.

**Study**	**Participants (female)**	**Country**	**Population**	**Mean age (years)**
Brenner et al. (30)	34 (2)	USA	Veterans with moderate to severe traumatic brain injury	51.6 (10.7)
Clore and Gaynor (34)	30 (22)	USA	Undergraduate students from a large American university who reported significant distress and low self-esteem	21.10 (5.11)
Czyz et al. (37)	36 (28)	USA	Adolescents hospitalized due to suicide risk	15.42 (1.36)
Franklin et al. (31)	114 (92) 131 (97) 163 (96)	USA	Participants recruited from online web forums on self-injury or psychopathology	23.02 (5.47) 22.91 (4.99) 24.5 (6.61)
Hooley et al. (32)	144 (123)	USA	Participants recruited from online forums related to self-injury and severe psychopathology	25.63 (5.83)
Jun et al. (41)	45 (22)	Korea	Hospitalized patients with mental illness	44.95 (15.54)
MacPherson et al. (38)	72 (30)	USA	Children with pediatric bipolar disorder	9.22 (1.59)
Pachankis et al. (39)	108 (76)	USA	Sexual minority young adults	23.68 (3.11)
Pratt et al. (35)	62 (0)	UK	Male prisoners	35.2 (11.10)
Randell et al. (36)	341 (Not reported)	USA	Students in grades 9–12	Not reported
Simpson et al. (33)	17 (Not reported)	Australia	Participants with severe traumatic brain injury with posttraumatic amnesia	41.88 (11.92)
Thompson et al. (40)	108 (58)	USA	High-risk youths in grades 9–12	15.86 (1.03)

### Intervention Characteristics

Individual study characteristics are reported in Table 2. The majority of studies focus on self-esteem as a treatment target, with others focusing on related self-concepts, including self-criticism, self-determination, self-efficacy, self-image, self-affirmation, and self-worth. Most of the studies used modified interventions based on existing techniques or theoretical models. Two studies used an intervention focused specifically on changing self-perception through writing (32, 39). One study used an online intervention based on a therapeutic evaluative condition to increase aversion toward suicidal behaviors and reduce self-criticism (31). One study used a safety planning and motivational interviewing approach based on self-efficacy and self-determination theory (37). One study compared the effectiveness of restructuring negative self-thoughts using thought record and strengthening positive self-statements using flashcard rehearsal (34). Other studies incorporated changing self-esteem as part of a treatment using either cognitive behavior therapy (30, 33, 35, 37, 41) or safety planning (36, 40).

**Table 2:** Intervention characteristics.

**Study**	**Theoretical model**	**Program outline self-esteem component**	**Self-concept**	**Duration**	**Assessment time points**	**Provider**	**Delivery method**	**Control group**	**Suicidal outcomes**	**Suicide scale**
Brenner et al. (30)	CBT	Program outline: Window to Hope program is based on principles and techniques from cognitive behavior therapy Self-esteem component: Session 9 “Building Hope” focuses on building self-esteem	Self-esteem	10 weekly 2 h sessions	Pre/3/6 months	Clinicians with doctoral degree in psychology or related field	F2F group	Waitlist control	Suicidal ideation	BSS
Clore and Gaynor (34)	CBT	The study compared two conditions (a) restructuring of negative self-thoughts (*via* training and daily practice using the Thought Record) and (b) enhancement of positive self-statements (*via* fluency training and daily flashcard rehearsal)	Self-esteem	Three weekly sessions. The first session lasted 2 h The second and third therapy sessions each lasted 1 h	Pre/post/5 weeks	The first author, a doctoral student in clinical psychology	F2F individual	Active control: The study compared between restructuring negative self-thoughts using thought record and enhancing positive self-statements through flashcard rehearsal.	Suicidal ideation	Items 9 and 39 from the Brief Symptoms Inventory and item 9 from the Beck Depression Inventory-II
Czyz et al. (37)	Safety planning and motivational interviewing	The full program is based on self-determination theory and self-efficacy theory.	Self-determination Self-efficacy	Not reported	Pre/1/3 months	First and second author	F2F Individual and family	TAU: Recovery action plan, including crisis management strategies and safety planning.	Suicidal ideation and attempts	C-SSRS
Franklin et al. (31)	Therapeutic evaluative condition	A main focus of the treatment is reducing aversion toward the self	Self-criticism	Participants can access the online treatment as desired over the course of 1 month	Pre/post/2 months	None	Online self-practice	Active: TEC, which displays neutral images	NSSI/suicidal ideation/suicide plan/suicidal behaviors	SITBI
Hooley et al. (32)	Writing interventions	Autobiographical Self-Enhancement Training (ASET) – a novel, cognitive intervention for NSSI focused on reducing self-criticism and enhancing positive self-worth	Self-criticism Self-worth	28 days. Participants write for 5 min each day	Pre/post/1/3 months	None	Online self-practice	Active: Expressive writing; Active: Daily journaling	NSSI/suicidal ideation/suicide plan/suicidal behaviors	SITBI
Jun et al. (41)	CBT	Session 2 focuses on establishing a positive self-image	Self-image	8–60 min sessions over 4 weeks	Pre/post	A psychiatric mental health Advanced Practice Nurse	F2F Group (4–5 patients per group)	TAU: medications, activity therapies, and supportive counseling by doctors and nurses	Suicide ideation	BSS
MacPherson et al. (38)	Child and family focus -CBT	A manualized, family-based intervention CBT with psychoeducation and complementary mindfulness and interpersonal techniques. The component “I can do it” focuses on improving children's self-esteem and parents' self-efficacy	Self-esteem	12 weekly 60–90 min sessions	Pre/4/8/12/39 weeks	Pre- and post-doctoral trainees in clinical psychology, who are novice at PBD treatment	F2F Family	TAU: TAU sessions were not manipulated in terms of content or structure	Suicide behaviors NSSI	C-SSRS Non-suicidal physical self-damaging Acts
Pachankis et al. (39)	Writing intervention	Writing intervention using self-affirmation prompts	Self-affirmation	Up to three sessions across three consecutive days	Pre/post/3 months	None	Online self-practice	Active: expressive writing prompts; active: control writing prompt (write about daily activities)	Suicidal ideation	Suicidal ideation attributes scale
Pratt et al. (35)	Cognitive-behavioral suicide prevention therapy	One of the five component focus on improving self-esteem and positive schema	Self-esteem	Up to 20 1-h sessions, delivered twice weekly during the initial phases and once weekly when therapeutic engagement had been established	Pre/4/6 months	Clinical psychologists (doctoral level) with 2–5 years of experience delivering CBT	F2F individual	TAU: received the usual care and support available	NSSI/suicidal ideation/suicide potential	BSS suicide probability scale
Randell et al. (36)	Life skills training	Life skills training program CAST (coping and support training) Provide specific skills training in building self-esteem	Self-esteem	12 1-h sessions over 6 weeks	Pre/4/10 weeks	Specifically trained group leaders	F2F Group (6–7 students per group)	Active: C-CARE Experimental Condition (comprehensive assessment of risk and protective factors and a brief motivational counseling intervention); TAU	Suicidal behaviors	The high school questionnaire
Simpson et al. (33)	CBT	Window to Hope program (WtoH) based on principles and techniques of CBT. Session 9 “Building Hope” focuses on building self-esteem	Self-esteem	10 weekly 2-h sessions	Pre/post/3 months	Therapist	F2F Group (2 people per group)	Waitlist control	Suicidal ideation	BSS
Thompson et al. (40)	Social support and life skills training	Personal Growth Class (PGC) One of the elements is, life-skills training, focusing on four core program components (self-esteem enhancement; decision making; anger, depression, and stress management; and interpersonal communication)	Self-esteem	Daily 55-min sessions from 18 to 36 weeks	Pre/post/5 months	Trained school personnel (e.g., teacher, counselor, or school nurse) who acted as group leaders	F2F Group (12 students per group)	Assessment only	Suicide risk behaviors	Suicide risk behaviors scale

*BSS, Beck Scale for Suicide Ideation; CBT, Cognitive behavior therapy; C-SSRS, Columbus-Suicide Severity Rating Scale; F2F, Face to face; NSSI, Non-suicidal self-injury; PBD, Pediatric bipolar disorder; SITBI, Self-injurious thoughts and behaviors interview; TAU, Treatment as usual*.

All studies were RCTs with nine (30, 31, 33–35, 37, 38, 40, 41) using a two-arm design and three (32, 36, 39) using a three-arm design. In terms of the control group, two studies (30, 33) compared the treatment with a wait-list control, four (35, 37, 38, 41) used treatments as usual, four (31, 32, 34, 39) used an active control, one (40) used assessment only, and one (36) used both active and treatment as usual. In terms of delivery method, five studies (30, 33, 36, 40, 41) reported that treatments were delivered in groups, two studies (34, 35) delivered treatment to individuals, three studies (31, 32, 39) used self-practice, one (38) was implemented as a family intervention, and one (37) combined both family and individual sessions. Additionally, in nine studies (30, 33–38, 40, 41) the intervention was delivered face to face and in three (31, 32, 39) it was delivered online. Treatment ranged from 3 to 36 sessions, with the sessions in most studies delivered one or twice weekly. Sessions typically lasted for 1 h. Follow-up timepoints were reported in 11 studies and varied from 4 week to 39 weeks.

Different suicidal behaviors were used in the included studies, with some reporting multiple suicidal outcomes. Specifically, nine studies measured suicidal ideation, four reported results for NSSI, five reported suicidal behaviors, two reported suicide plan, one reported suicide attempt, and one reported suicide potential. Most studies measured suicidality using the Beck Scale for Suicide Ideation (42). Other measurements such as Columbia-Suicide Severity Rating Scale (43) or Self-Injurious Thoughts and Behaviors Interview (44), were also reported.

### Quality of Included Studies

**Figure 2:**
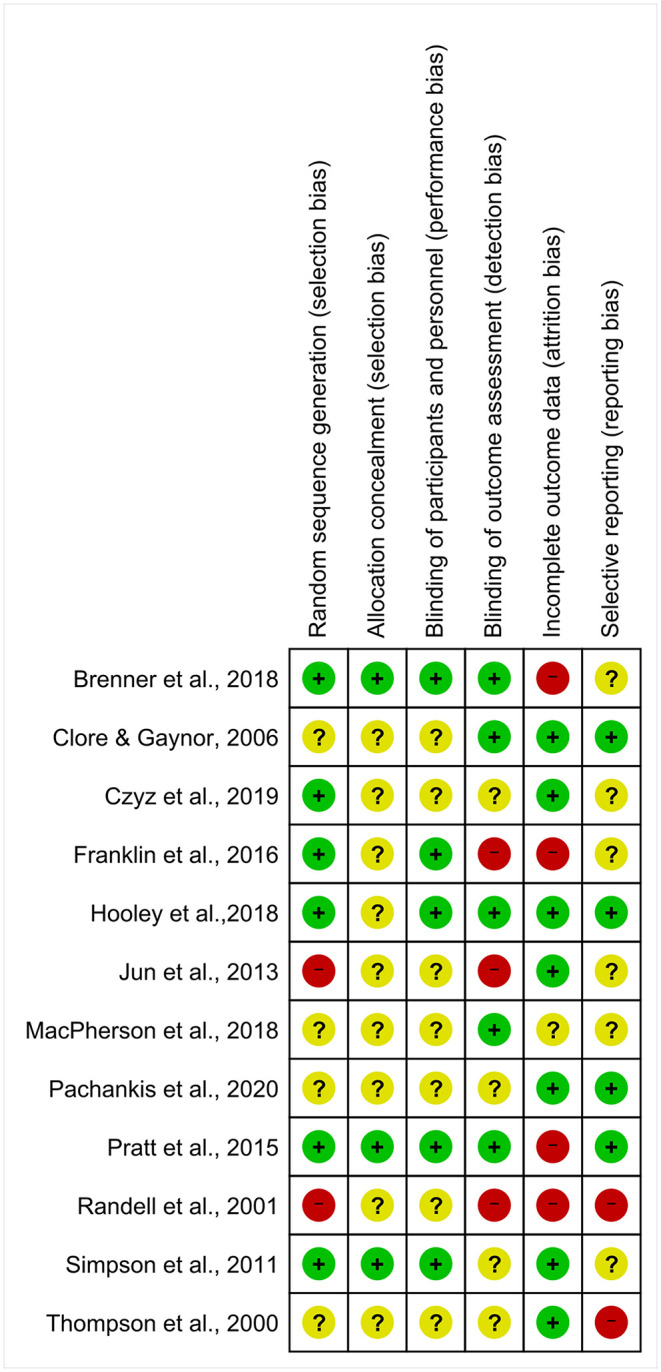
Risk of bias summary.

The overall evaluation of risk of bias is reported in Figure 2. Sequence generation method was described in detail and appropriately used in five studies, was unclear in four studies and was evaluated as high risk in two studies. Allocation concealment had the highest proportion of unclear risk of bias except for three studies where it was rated as low risk. Blinding of participants and personnel also showed high unclear risk of bias, with five studies rated as low risk. In terms of blinding of outcome assessment, five studies were evaluated as low risk, three as high risk and four as unclear. Risk of bias for incomplete outcome data was evaluated in most studies, with seven studies rated as low risk, four as high risk, and only one was rated as unclear. For selective reporting, three studies published a protocol and thus we were able to compare and confirm their outcomes. One study (34) reported each outcome in detail, so it was also rated as low risk. The other eight studies were rated as high risk. Hooley et al.'s study had the lowest proportion of risk of bias with five out of six domains rated as low risk (32). Randell et al.'s (36) study appears to be the weakest with four domains rated as having high risk of bias. MacPherson et al.'s (38) study had the most unclear risk of bias with five domains rated as unclear. Detailed comments on authors' evaluations of risk of bias for each study are available in Appendix B.

### Overview of Meta-Analyses on Outcome Measures

Necessary data for six studies (32, 35–38, 40) were not available and one study (34) used comparisons between two self-esteem conditions; thus, these studies were excluded from the meta-analysis. Additionally, Franklin et al. (31) reported the data for suicidal behaviors as the sum of suicidal behavior frequencies during each week of the treatment and post-treatment month; thus, their study was also excluded from the meta-analysis. The corresponding authors of the six papers that did not include the necessary data for meta-analysis were contacted *via* email and requested to provide these data. Hooley et al. (32) replied and the data from their study were included in the analysis. The other five did not respond and their studies were excluded from the meta-analysis, though not from the systematic review.

Only data for suicidal ideation was available for the meta-analysis. Follow-up data were available for most studies included in the meta-analysis but with different follow-up timepoints. Following Cochrane's recommendation (29), data at 3-month-follow-up for suicidal ideation was extracted for the meta-analysis.

### Effects on Suicidal Ideation at Post Intervention and 3-Month Follow-Up

**Figure 3:**
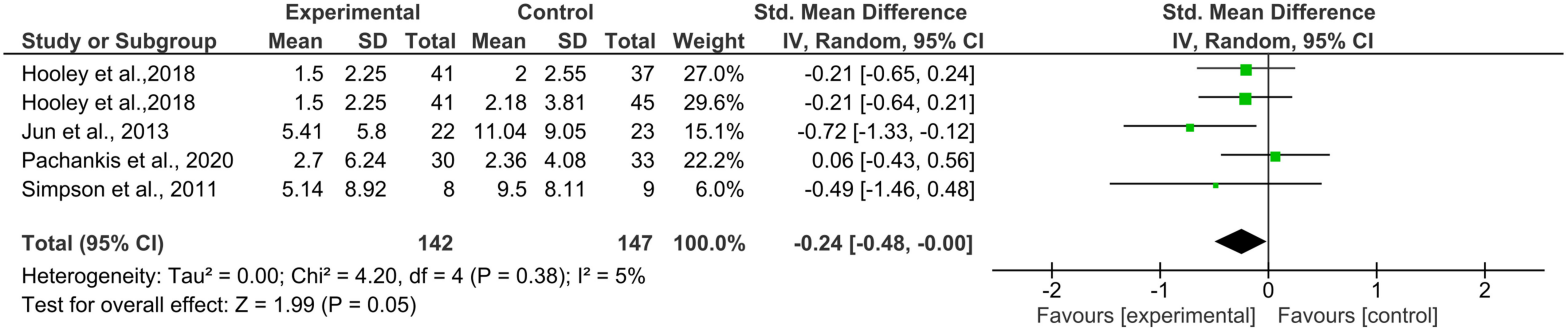
Effects on suicidal ideation at post-intervention.

A total of five comparisons (*N* = 289) from four studies (32, 33, 39, 41) compared a self-esteem-related intervention to a control group at post-intervention. Results revealed that compared with the control condition, the participants in the self-esteem-related interventions experienced a small but significant reduction in suicidal ideation [*g* = −0.24, 95% CI (−0.48, 0.00); Figure 3]. The heterogeneity between groups was non-significant and small (*I*^2^ = 5%, *p* = 0.38).

**Figure 4:**
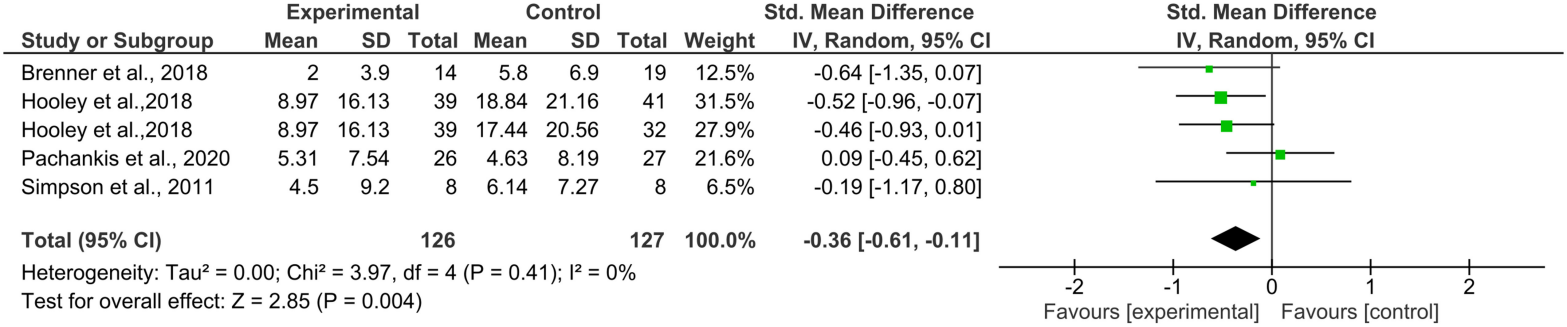
Effects on suicidal ideation at 3-month follow-up.

Data for 3-month follow-up for suicidal ideation were available from four studies (30, 32, 33, 39) with five comparisons (*N* = 253). The meta-analysis at 3-month follow-up also yielded a small but significant effect size [*g* = −0.36, 95% CI (−0.62, −0.11); Figure 4], showing that the treatment condition was more effective at reducing suicidal ideation. Heterogeneity was low and non-significant (*I*^2^ = 0%, *p* = 0.41).

## Discussion

To our knowledge, this is the first systematic review and meta-analysis investigating the effectiveness of self-esteem-related interventions in reducing suicidal behaviors. The database search yielded 12 RCTs studies that used a self-esteem related intervention for suicidality. Among them, only five studies provided sufficient data for the meta-analysis. The results of the meta-analysis revealed that self-esteem-related interventions have a small but significant effect in reducing suicidal ideation at post intervention and 3-month follow-up.

The effect size for suicidal ideation [g = −0.24, 95% CI (−0.48, 0.00)] at post-intervention from the current analysis was compared with similar meta-analysis involving other types of interventions. The results suggest that self-esteem-related interventions could be more effective than self-guided digital interventions [g = −0.18, 95% CI (−0.27, −0.10)] (45), as effective as face-to-face CBT [g = −0.24, 95% CI (−0.41, −0.07)] (46), but less effective than dialectical behavior therapy [g = −0.31, 95% CI (−0.52, −0.09)] (47) in reducing suicidal ideation post intervention.

The results revealed that the included studies used diverse intervention methods. Treatment ranged from CBT to writing intervention, with only three studies (32, 34, 39) using interventions focused specifically on enhancing self-esteem. Additionally, different therapies have different approaches to self-esteem enhancement. For example, it is suggested that CBT can enhance self-esteem by encouraging individuals to become aware of their own negative predictions and self-critical thoughts, and then reconstruct their negative self-image by challenging and testing the predictions and thoughts (48). For writing interventions, it is believed that writing helps in organizing and altering the way an event is presented in memory, allowing individuals to recall more dimensions of the event and thus helping them have a more objective view of themselves (49). In evaluative conditioning, the way an individual views themselves is restructured by repeatedly pairing words that represent the self (i.e., I or me) with positively valenced stimuli (i.e., positive words or images) (31, 50).

It has been suggested that enhancing self-esteem is effective in the prevention of suicide, as high self-esteem could buffer the negative effects of many psychological risk factors for suicide (23). Specifically, self-esteem has been shown to be a protective factor against several suicide risk factors, namely depression (51, 52), anxiety (52, 53), loneliness (52), and hopelessness (54). Thus, it is possible that when an intervention incorporates or focuses exclusively on self-esteem enhancement, the heightened sense of self-esteem could buffer against the negative impact of risk factors for suicide. The interpersonal theory of suicide offers another possible explanation for how increasing self-esteem might lead to a decrease in suicidal behaviors (55). The theory posits that suicidal desire results from the co-occurrence of thwarted belongingness (i.e., feelings that one is disconnected from others) and perceived burdensomeness (i.e., feelings that one is a burden on others). As self-hate is an indicator of perceived burdensomeness, it is likely that increasing self-esteem helps to alleviate the feelings of being a burden on others and lead to a reduction in suicidal behaviors.

A somewhat surprising finding from the current review is that the effect size for suicidal ideation at the 3-month follow-up was higher than at post-intervention. This result demonstrates that the effect on suicidal ideation caused by self-esteem-related interventions could be maintained even at follow-up. A possible explanation for this result is that the main goal of self-esteem programs is targeting and modifying self-critical thinking (56). Since self-critical thinking has been shown to play an important role in the development and maintenance of psychopathology, it is likely that a program with self-esteem component could lead to long-term maintenance of reduction in suicidal ideation (56, 57).

### Limitations

Although these results show a potential for self-esteem-related interventions in reducing suicide, they should be interpreted with caution due to several limitations. The numbers of included papers in this meta-analysis were small with a small sample size and varied sample population, which could lead to a high risk of bias. Different suicidal outcomes and follow-up periods also made it difficult to calculate the pooled effect size. Further, unlike previous studies (11, 24, 25), the current systematic review and meta-analysis limited the definition of self-esteem intervention to only those studies that incorporate self-esteem as one of the treatment components. However, it must be noted that most of the studies included in the review not only include self-esteem enhancement but also other treatment modules. This makes it difficult to isolate self-esteem as the cause of reduction in suicidal behaviors. Nevertheless, we used broad inclusion criteria because, so far, there are few interventions that focus specifically on enhancing self-esteem. Furthermore, the Cochrane guidelines for the systematic review of intervention studies also suggest that reviews with broad inclusion criteria are feasible and encouraged because the results could provide clues regarding whether an intervention operates differently in certain conditions (in this case, whether self-esteem-related intervention is effective in reducing suicide) (29). Some of the included studies involve high risk of bias in certain domains, especially in blinding of outcome and incomplete outcome data. Lastly, the current review and meta-analysis was unable to control for potential confounding factors, including age and follow-up periods. While trajectory of self-esteem has been shown to change over time (13), we were unable to separate data between the adolescent group and the adult group in the current review due to the small number of studies. Similarly, given the enduring nature of suicide (55), studies with longer follow-up periods are needed to confirm these results. Thus, our conclusion must be interpreted with caution because of methodological limitations of analyses.

### Implications for Practice

While self-esteem has been demonstrated to play a crucial role in mental health and well-being, self-esteem-based interventions remain nascent. As high self-esteem could protect against suicidality, it is possible that interventions for suicide may improve treatment efficacy by focusing exclusively on enhancing self-esteem or at least incorporate self-esteem enhancement as part of their treatment targets. Furthermore, while some self-esteem interventions or related psychological models currently exist (e.g., the intervention program “Everybody's Different”; the CBT model for low self-esteem) (58, 59), current evidence for these types of interventions is limited. Thus, new interventions that focus specifically on self-esteem or are established on self-esteem theory should be developed and evaluated. Additionally, as self-esteem has been suggested to be susceptible to change during young age (11, 12), it is possible that integrating self-esteem improvement into school curriculum may help to prevent future suicidal behaviors.

## Conclusion

In summary, this is the first systematic review and meta-analysis on the effects of self-esteem-related interventions in reducing suicidal behaviors. The findings suggest that self-esteem-related interventions are effective in reducing suicidal ideation at post intervention and 3-month follow-up. However, due to the broad inclusion criteria and limited number of studies, these results should be interpreted with caution. Moderators such as intervention length, delivery method, and the contents of intervention should be investigated in future review. Finally, future studies should also assess the long-term benefits and mechanisms of change for each type of intervention.

## Data Availability Statement

The raw data supporting the conclusions of this article will be made available by the authors, without undue reservation.

## Author Contributions

Conceptualization: NM. Study design, literature searches, studies screening, and visualization: NM and ND. Data analysis and writing—original draft: ND. Writing—review and editing: ND, NM, YK, SA, YF, KTo, KTa, and IK. Supervision and final script validation: NM, YK, SA, YF, KTo, KTa, and IK. All authors contributed to the article and approved the submitted version.

## Funding

This study was supported by Japan Society for Promotion of Science (JSPS) KAKENHI (to SA) (Grant Number: JP 18K0758308).

## Conflict of Interest

The authors declare that the research was conducted in the absence of any commercial or financial relationships that could be construed as a potential conflict of interest.

## Publisher's Note

All claims expressed in this article are solely those of the authors and do not necessarily represent those of their affiliated organizations, or those of the publisher, the editors and the reviewers. Any product that may be evaluated in this article, or claim that may be made by its manufacturer, is not guaranteed or endorsed by the publisher.
