# Supplementary material for: The Effectiveness of Self-Esteem-Related Interventions in Reducing Suicidal Behaviors: A Systematic Review and Meta-Analysis
Source: Front Psychiatry. 2022 Jun 15;13:925423. doi: 10.3389/fpsyt.2022.925423 (PMC9240430; doi:10.3389/fpsyt.2022.925423)
Supplement: Supplementary file 1 [file Table_1.DOCX]

**Appendix A. Search strategies**

| **Concept** | **Search terms** |
| --- | --- |
| Self-esteem | self-esteem/self-perception*/self-identit*/self-crit*/self-attack*/self-image/self-worth/self-efficacy |
| Intervention | intervention*/program*/ lesson/treatment*/psychoeducation/psychotherapy |
| Suicide | suicid* |

| **Database** | **Items found**  **(29 May 2021)** | **Items found**  **(4 April 2022)** |
| --- | --- | --- |
| **PubMed** |  |  |
| (("self-concept" [MESH] OR "self-concept" OR "self-esteem" OR "self-perception*" OR "self-identit*" OR "self-crit*" OR "self-attack*" OR "self-worth" OR "self-efficacy" OR "self-image") AND ("intervention*" OR "program*" OR "lesson*"OR "treatment*" OR "psychoeducation" OR "psychotherapy") AND ("Suicide"[Mesh] OR "Suicide, Attempted"[Mesh] OR suicid*)) | 1320 | 1420 |
| **PsycINFO** |  |  |
| (MeSH: "self-concept" OR Any Field: "self-concept" OR Any Field: "self-esteem" OR Any Field: "self-perception*" OR Any Field: "self-identit*" OR Any Field: "self -crit*" OR Any Field: "self-attack*" OR Any Field: "self-worth" OR Any Field: "self-efficacy" OR Any Field: "self-image" ) AND (Any Field: "intervention*" OR Any Field: "program*" OR Any Field: "lesson*" OR "treatment*" OR Any Field: "psychoeducation" OR Any Field: "psychotherapy") AND (MeSH: "Suicide" OR MeSH: "Suicide, Attempted" OR MeSH: suicidal ideation OR Any Field: suicid*) | 796 | 854 |
| **Web of Science** |  |  |
| ((ALL=("self-esteem" OR "self-concept" OR "self-perception*" OR "self-identit*" OR "self-crit*" OR "self-attack*" OR "self-image" OR "self-worth" OR "self-efficacy")) AND ALL=("intervention*" OR "program*" OR "lesson*" OR "treatment*" OR "psychoeducation" OR "psychotherapy")) AND ALL=(suicid*) | 1320 | 1436 |
| **Clinicaltrials.gov** |  |  |
| ("self-esteem" OR "self-concept" OR "self-perception" OR "self-identity" OR "self-criticism" OR "self-attack" OR "self-worth" OR "self-efficacy" OR "self-image") AND (suicide OR "suicidal ideation" OR "attempt suicide") | 188 | 204 |
