# Supplementary material for: The Effectiveness of Self-Esteem-Related Interventions in Reducing Suicidal Behaviors: A Systematic Review and Meta-Analysis
Source: Front Psychiatry. 2022 Jun 15;13:925423. doi: 10.3389/fpsyt.2022.925423 (PMC9240430; doi:10.3389/fpsyt.2022.925423)
Supplement: Supplementary file 2 [file Table_2.DOCX]

**Appendix B. Risk of Bias**

| **Bias** | **Authors' judgment** | **Support for judgment** |
| --- | --- | --- |
| **Brenner et al., 2018** | | |
| Random sequence generation (selection bias) | Low risk | A block randomization was used: when a group of 4 participants had been enrolled, they were randomly allocated to a condition using ordered sealed envelopes. |
| Allocation concealment (selection bias) | Low risk | Allocation to the study arms was conducted by the study coordinator, and allocation was concealed. A block randomization was used: when a group of 4 participants had been enrolled, they were randomly allocated to a condition using ordered sealed envelopes. |
| Blinding of participants and personnel (performance bias) | Low risk | Parallel group randomized controlled crossover trial with blind random allocation to the 2 arms on a 1:1 ratio. |
| Blinding of outcome assessment (detection bias) | Low risk | Both the Time 2 and Time 3 assessments were administered by an independent assessor who was blinded to the participants’ group assignment. Participants were reminded not to disclose their treatment condition to the blind assessor, and any inadvertent disclosures were noted. |
| Incomplete outcome data (attrition bias) | High risk | Dropout rates were different for Waitlist (from 22 to 15) and Active group (from 22 to 20) |
| Selective reporting (reporting bias) | Unclear risk | Could not find protocol |
| **Clore & Gaynor, 2006** | | |
| Random sequence generation (selection bias) | Unclear risk | Participants were stratified by gender and then randomized. |
| Allocation concealment (selection bias) | Unclear risk | None |
| Blinding of participants and personnel (performance bias) | Unclear risk | None |
| Blinding of outcome assessment (detection bias) | Low risk | To reduce potential demand characteristics, participants were informed that during the treatment part of the study the experimenter was kept blind to all measures except for those used in determining eligibility and implementing the initial portion of the intervention). |
| Incomplete outcome data (attrition bias) | Low risk | Seven dropouts after the first treatment session and three following the second treatment session. Attrition rates were comparable in both conditions: five dropped out from FT and five from the TR condition. When provided, reasons for dropping out of the study included family emergencies, other commitments, and seeking therapy elsewhere. Dropouts did not differ significantly from completers on the RSES, F(1, 30) = 0.60, *p* = 0.45, or the BSI-GSI, F(1, 30) = 0.43, *p* = 0.5 |
| Selective reporting (reporting bias) | Low risk | Outcome data and methods used to deal with missing data were thoroughly reported. |
| **Czyz et al., 2019** | | |
| Random sequence generation (selection bias) | Low risk | Following baseline assessment, participants were randomized to either TAU (n = 18) or TAU plus intervention group (MI-SafeCope; n = 18) using a sequential treatment assignment system based on an approach described in Pocock and Simon (1975) and available through Consulting for Statistics, Computing and Analytics. Randomization was stratified based on gender and multiple suicide attempt history, which produced balanced group assignments. |
| Allocation concealment (selection bias) | Unclear risk | None |
| Blinding of participants and personnel (performance bias) | Unclear risk | None |
| Blinding of outcome assessment (detection bias) | Unclear risk | None |
| Incomplete outcome data (attrition bias) | Low risk | There were only three dropouts in the intervention group and one in the TAU group |
| Selective reporting (reporting bias) | Unclear risk | None |
| **Franklin et al., 2016** | | |
| Random sequence generation (selection bias) | Low risk | Participants were randomly assigned via a random sequence generator. |
| Allocation concealment (selection bias) | Unclear risk | None |
| Blinding of participants and personnel (performance bias) | Low risk | The informed consent form clearly stated the treatment-related aspects of the study but did not provide details about TEC that would have allowed participants to discern whether or not they were in the active or control group. No person administered the study; it was administered through an app. |
| Blinding of outcome assessment (detection bias) | High risk | Participants self-reported the outcomes. |
| Incomplete outcome data (attrition bias) | High risk | Data from baseline dropped to half at post treatment for both groups (Treatment group baseline n=75 to posttreatment n=33; Control group baseline n=84 to posttreatment n=48). |
| Selective reporting (reporting bias) | Unclear risk | There is no protocol. |
| **Hooley et al., 2018** | | |
| Random sequence generation (selection bias) | Low risk | Used randomization software within Qualtrics. |
| Allocation concealment (selection bias) | Unclear risk | None |
| Blinding of participants and personnel (performance bias) | Low risk | Participants did not know about the other conditions, although the key people from the study team knew who was assigned to each condition. However, the assessments were not made by anyone from the study team (Information received upon emailing the study team). |
| Blinding of outcome assessment (detection bias) | Low risk | Participants were simply asked to complete study questionnaires on a regular basis. The assessments were not made by anyone from the study team (Information received upon emailing the study team). |
| Incomplete outcome data (attrition bias) | Low risk | Data were missing due to drop out; missing outcome data similar in numbers across intervention groups; plausible effect size among missing outcomes may not be sufficient to exert a clinically relevant impact on observed effect size. |
| Selective reporting (reporting bias) | Low risk | Outcomes were the same with the protocol (Protocol ID: ISRCTN12276176) |
| **Jun et al., 2013** | | |
| Random sequence generation (selection bias) | High risk | Patients who were hospitalized in May and June were assigned to the control group, and patients who were hospitalized in August and September were assigned to the experimental group. |
| Allocation concealment (selection bias) | Unclear risk | None |
| Blinding of participants and personnel (performance bias) | Unclear risk | None |
| Blinding of outcome assessment (detection bias) | High risk | Self-reported outcomes. No blinding of outcome assessment, and the outcome measurement is likely to be influenced by lack of blinding (outcome was related to suicide). |
| Incomplete outcome data (attrition bias) | Low risk | A total of five participants (three in the experimental group and two in the control group) dropped out because they were discharged from the hospital before the end of the program. |
| Selective reporting (reporting bias) | Unclear risk | None |
| **MacPherson et al., 2013** | | |
| Random sequence generation (selection bias) | Unclear risk | Children with PBD subsequently completed the baseline assessment and were randomized to treatment condition (but no randomization method was mentioned). |
| Allocation concealment (selection bias) | Unclear risk | None |
| Blinding of participants and personnel (performance bias) | Unclear risk | None |
| Blinding of outcome assessment (detection bias) | Low risk | All the important assessments (i.e., suicide, NSSI) were conducted using structured interviews. Additional evaluations were conducted at 4-, 8-, 12- (posttreatment), and 39-weeks (6-month follow-up) by blinded raters |
| Incomplete outcome data (attrition bias) | Unclear risk | None |
| Selective reporting (reporting bias) | Unclear risk | None |
| **Pachankis et al., 2020** | | |
| Random sequence generation (selection bias) | Unclear risk | The method section of the paper mentioned randomization. However, the methods were not clear. |
| Allocation concealment (selection bias) | Unclear risk | None |
| Blinding of participants and personnel (performance bias) | Unclear risk | None |
| Blinding of outcome assessment (detection bias) | Unclear risk | None |
| Incomplete outcome data (attrition bias) | Low risk | Dropout rate was similar between the control (from n=36 to n=27) and self-affirmation (from n=36 to n=26) groups |
| Selective reporting (reporting bias) | Low risk | Outcome report exactly with the protocol (Clinicaltrials.gov NCT03751020) |
| **Pratt et al., 2015** | | |
| Random sequence generation (selection bias) | Low risk | Randomization of participants to the two treatment groups was achieved by referring to a sequence of sealed envelopes provided by the Research Statistician (GD). Treatment allocated was based on pseudorandom number generation, and based on randomly-permuted blocks algorithm (with block sizes randomly varying between 4 and 8) |
| Allocation concealment (selection bias) | Low risk | Randomization of participants to the two treatment groups was achieved by referring to a sequence of sealed envelopes provided by the Research Statistician (GD). Treatment allocated was based on pseudorandom number generation, and based on randomly-permuted blocks algorithm (with block sizes randomly varying between 4 and 8).  The randomization schedules were generated and provided to the study by the Research Statistician, before being kept securely and confidentially by the Trial Administrator who contacted the trial therapists, as appropriate, with the participant's details for those allocated to the CBSP group. Thus, randomization was independent and the research assistants completing the assessments were blind to group allocation. A number of strategies were developed to achieve and maintain the masking of assessors, such as removing any research assistant involvement in the random assignment process, research assistant and trial therapist to avoid simultaneous use of allocated interview/therapy rooms to preserve blindness to allocation, and participants were encouraged at each assessment not to refer to treatment group allocation. |
| Blinding of participants and personnel (performance bias) | Low risk | The study was a single-blind (rater) randomized controlled pilot trial. |
| Blinding of outcome assessment (detection bias) | Low risk | Outcomes were assessed by examining the prisoners’ data. (In accordance with the trial protocol, the primary outcome measure was the number of episodes of suicidal or self-injurious behavior (SIB) in the past six months assessed by examination of participants’ prison records). Additionally, the rater was blinded (the PROSPeR study was a single-blind (rater) randomized controlled pilot trial). |
| Incomplete outcome data (attrition bias) | High risk | Dropout rates were high. The follow up rates for the study sample as a whole was 40 out of 62 (65%) at 4 months and 35 (56%) at the 6 months assessments. Five (8%) participants withdrew from the study and we were unable to follow-up 22 (35%) participants who had been unexpectedly released early or transferred to other prisons for security reasons during the course of the trial. Participants that were lost to follow up did not differ significantly from participants that completed the 4 months or 6 months assessment on any of the socio-demographic or custodial characteristics. |
| Selective reporting (reporting bias) | Low risk | The PROSPeR study was registered as an International Standard Randomised Controlled Trial, ISRCTN59909209. Same outcomes as protocol. |
| **Randell et al., 2001** | | |
| Random sequence generation (selection bias) | High risk | Sampling involved random assignment by school to one of the two groups. |
| Allocation concealment (selection bias) | Unclear risk | None |
| Blinding of participants and personnel (performance bias) | Unclear risk | None |
| Blinding of outcome assessment (detection bias) | High risk | Each group leader coded their videotape after each session and rated each student’s demonstration of skills acquired. |
| Incomplete outcome data (attrition bias) | High risk | In the C-CARE and control groups, those who did not complete had slightly lower, but significant, initial levels of the outcome measures. |
| Selective reporting (reporting bias) | High risk | Unclear outcome reporting; lack of information about intervention effects. |
| **Simpson et al., 2011** | | |
| Random sequence generation (selection bias) | Low risk | A block randomization procedure was used: When a group of 4 participants was enrolled, they were randomly allocated to a condition using a computer-generated set of random numbers. All participants remained in the group to which they had been allocated |
| Allocation concealment (selection bias) | Low risk | Allocation to the study arms was conducted off-site by the second author (RT) and allocation was concealed. |
| Blinding of participants and personnel (performance bias) | Low risk | Assessments at Time 2 and Time 3 used an independent assessor who was blinded to the condition. Participants were reminded not to disclose their treatment condition to the assessor. To check the success of blinding, the assessor was asked to record any inadvertent disclosure by participants of their treatment arm and to also guess whether the person was in the treatment or wait-list group. One person was unblinded at Time 2 (self-disclosure) and was, therefore, unblinded at Time 3. It is noted that author GKS conducted the Time 1 (baseline) testing and delivered the therapy program. However, because randomization occurred after the Time 1 (baseline) testing, GKS was effectively blind to group allocation. |
| Blinding of outcome assessment (detection bias) | Unclear risk | None |
| Incomplete outcome data (attrition bias) | Low risk | Only one person dropped out from the waitlist group (prior to treatment) but the data were not included in the analysis |
| Selective reporting (reporting bias) | Unclear risk | Could not find protocol. |
| **Thompson et al., 2000** | | |
| Random sequence generation (selection bias) | Unclear risk | None |
| Allocation concealment (selection bias) | Unclear risk | None |
| Blinding of participants and personnel (performance bias) | Unclear risk | None |
| Blinding of outcome assessment (detection bias) | Unclear risk | None |
| Incomplete outcome data (attrition bias) | Low risk | Across time, retention rates for study participants were fairly equal across the three groups. |
| Selective reporting (reporting bias) | High risk | Unclear outcome reporting; lack of information about intervention effects. |
